# Dynamic Reconfiguration of Subcompartment Architectures in Artificial Cells

**DOI:** 10.1021/acsnano.2c02195

**Published:** 2022-06-13

**Authors:** Greta Zubaite, James W. Hindley, Oscar Ces, Yuval Elani

**Affiliations:** † Department of Chemistry, Molecular Sciences Research Hub, Imperial College London, 82 Wood Lane, London W12 0BZ, United Kingdom; ‡ Institute of Chemical Biology, Molecular Sciences Research Hub, Imperial College London, 82 Wood Lane, London W12 0BZ, United Kingdom; § fabriCELL, Molecular Sciences Research Hub, Imperial College London, 82 Wood Lane, London W12 0BZ, United Kingdom; ∥ Department of Chemical Engineering, Imperial College London, Exhibition Road, London SW7 2AZ, United Kingdom

**Keywords:** artificial cells, phospholipids, compartments, organelles, vesicles

## Abstract

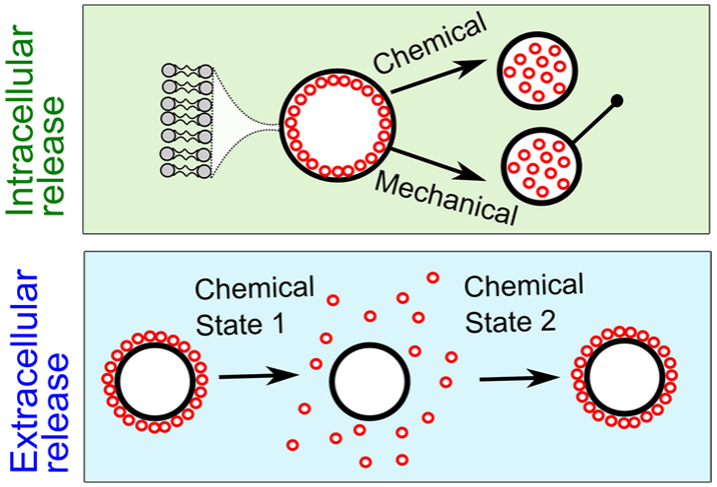

Artificial cells are minimal structures constructed from biomolecular building blocks designed to mimic cellular processes, behaviors, and architectures. One near-ubiquitous feature of cellular life is the spatial organization of internal content. We know from biology that organization of content (including in membrane-bound organelles) is linked to cellular functions and that this feature is dynamic: the presence, location, and degree of compartmentalization changes over time. Vesicle-based artificial cells, however, are not currently able to mimic this fundamental cellular property. Here, we describe an artificial cell design strategy that addresses this technological bottleneck. We create a series of artificial cell architectures which possess multicompartment assemblies localized either on the inner or on the outer surface of the artificial cell membrane. Exploiting liquid–liquid phase separation, we can also engineer spatially segregated regions of condensed subcompartments attached to the cell surface, aligning with coexisting membrane domains. These structures can sense changes in environmental conditions and respond by reversibly transitioning from condensed multicompartment layers on the membrane surface to a dispersed state in the cell lumen, mimicking the dynamic compartmentalization found in biological cells. Likewise, we engineer exosome-like subcompartments that can be released to the environment. We can achieve this by using two types of triggers: chemical (addition of salts) and mechanical (by pulling membrane tethers using optical traps). These approaches allow us to control the compartmentalization state of artificial cells on population and single-cell levels.

In recent decades there has been growing interest in developing artificial cells that possess desired functions and behaviors that are easier to predict and control than those of naturally occurring biological cells.^1−3^ This bottom-up approach to synthetic biology involves constructing artificial cells using well-characterized biomolecular building blocks. Artificial cells can be used as cell models to study fundamental biological processes and as programmable micromachines for industrial and clinical applications.^1,3−8^

The development of a fully functioning artificial cell that can replicate all key aspects of their living counterparts is considered one of the ultimate goals of synthetic biology. In particular, there have been significant efforts to recapitulate various architectural features of biological cells. Beyond simple biomimetics, a cell’s size, shape, and level of compartmentalization is tightly related to its behavior, performance, and capabilities;^9−11^ form and function are intertwined. For this reason, designing artificial cells which exhibit different levels of spatial organization is a particularly active research area.

Artificial cell compartmentalization has been used to mimic nuclei,^12−14^ for energy generation,^15^ carbon fixation,^16^ and metabolism.^17,18^ Compartmentalization has also been used as a design principle in responsive cell-mimetic microsystems, where internal processes are initiated following the breakdown/permeabilisation of compartments,^17,19,20^ in microreactor development^20−22^ and in recreating signaling cascades.^19^ Unlike in biological cells, the localization of vesicle-based subcompartments in artificial cells is static and nondynamic. Here, we describe higher order lipid membrane-based arrangements that allow control over the presence, location, and distribution of internal substructures, with the architectural arrangement modulated by the cell’s environment.

Subcompartmentalization is a ubiquitous feature in biological cells, and segregating cellular biochemical processes is crucial for optimal activity. Biological cells achieve this in various ways including through organelles and membraneless phase separated structures.^23−25^ Crucially, the spatial arrangements of cellular components are usually well-defined. Examples of this are interactions of specific organelle membrane sites with proteins or substrate pools in the cytoplasm^26^ or direct interactions between different organelles that are required for vital cellular functions.^27−29^ Various mechanisms are involved in enriching these contact sites with proteins and phospholipids required to maintain these organelle interactions.^30,31^ Changes in cell state (cellular stress, endoplasmic reticulum stress, and reactive oxygen species) can also influence cellular spatial organization, for example, through the formation of membraneless organelles.^28^

Through these and other examples, it is evident that (i) there are biological consequences to how cell compartments are arranged in space, (ii) that cellular subcompartmentalization is dynamic, often changing in response to endogenous and exogenous triggers, and (iii) that control of subcompartmentalization is vital for efficient cellular processes. In most artificial cell systems, however, where compartments do exist, they are uniformly dispersed in the cell lumen; spatial organization is uncontrolled and fixed, unlike the dynamic level of organization that cells possess. Developing artificial cell strategies that implement this functionality is key in order to mimic their biological counterparts more closely, and to create artificial cells with higher degrees of functionality with the incorporation of dynamic compartmentalized motifs.

In this work, we develop an artificial cell design strategy where the spatial organization of subcompartments can be modulated in response to the cell’s chemical environment or mechanical triggers. Our cells are based on nested vesicle assemblies, where subcompartments (and the artificial cell itself) are delineated by lipid membranes. The nested structure involves the encapsulation of many smaller vesicles (100 nm diameter) within giant ones (5–50 μm diameter). We engineer our system so that the arrangement of subcompartments can switch between a condensed state (where they are associated with the cell membrane) and a dispersed state (where they dissociate from the membrane and are freely diffusing). Our design strategy is underpinned by the electrostatic interactions between subcompartments and the cell membrane, which are oppositely charged.^32−34^ We also explore these interactions in phase separated artificial cells, where subcompartments associate with charged membrane domains. The presence of salt, which screens the attractive forces, is used to modulate the strength of the interaction. Using this principle, we can form an array of different multilayered architectures. Depending on the arrangement, subcompartments can be disassembled by increasing the outer solution salt concentration resulting in (i) controllable changes of inner compartment spatial localization or (ii) the reversible release of compartments into the environment. In the latter case, this yields a synthetic analogue of extracellular vesicles. Changing the valency of the salt used, we can control the response rate of assembly/disassembly, conferring another degree of control over the artificial cell compartment spatial organization. We can also achieve this response by applying mechanical stimuli: pulling tethers from the artificial cell membrane induces the dispersal of the inner subcompartment layer. These approaches allow us to create artificial cells that can respond to two different types of stimuli by changing their compartmentalization on a population and single-cell level.

## Results and Discussion

### Assembling Localized Multicompartment Structures for Artificial Cells

**Figure 1:**
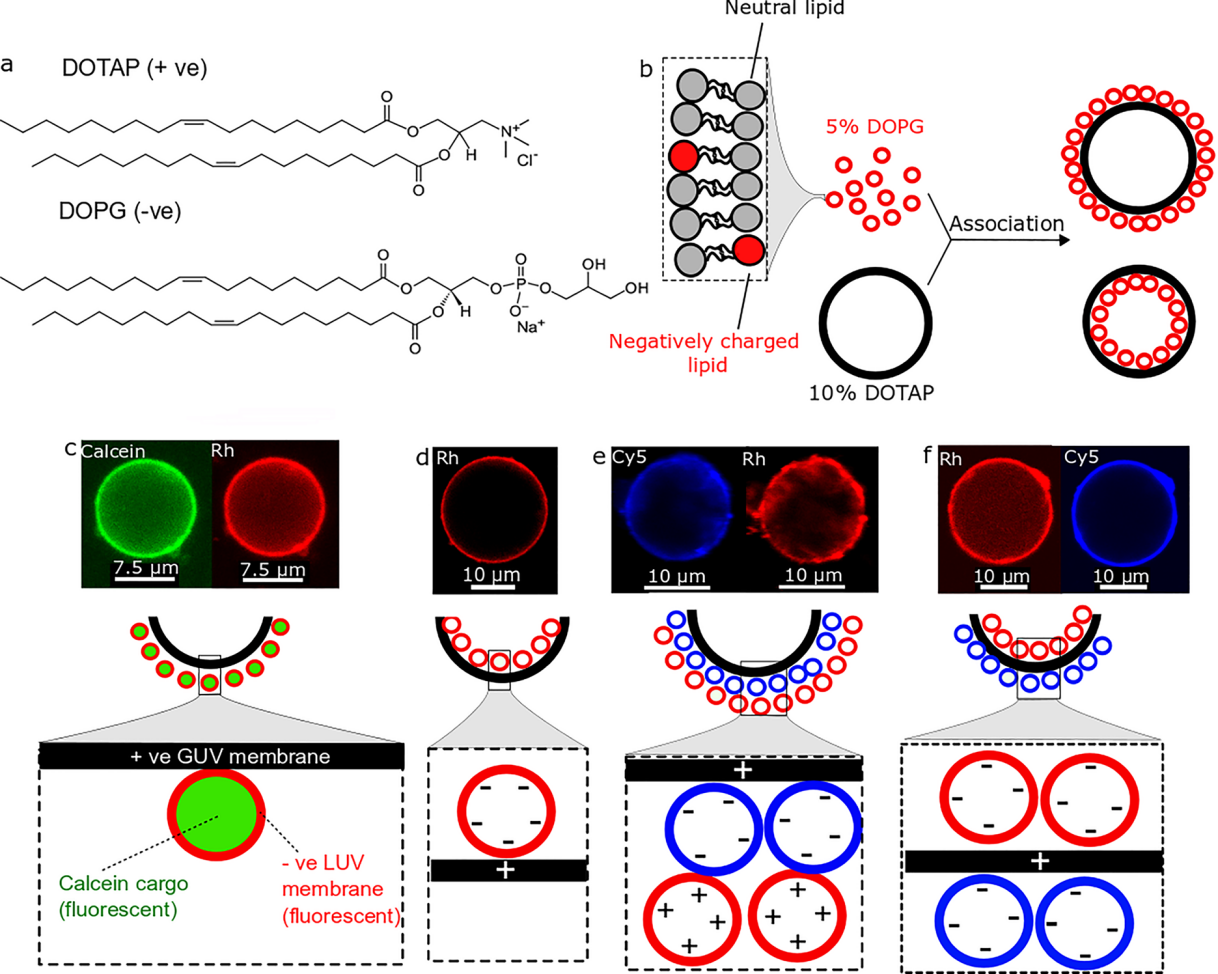
Generating artificial cells with different hierarchical architectures. (a) Chemical structures of charged phospholipids used. (b) Schematics of artificial cells with subcompartment layers composed of LUVs and GUVs. (c–f) Confocal microscopy fluorescence images and schematic representations of different hierarchical architectures achieved by forming multicompartment layers on oppositely charged artificial cell membranes. (c) Negatively charged LUV compartments layering on the outer surface of a positively charged GUV. Subcompartments are loaded with calcein dye and have a 1% Rh-PE fluorescently labeled membrane. (d) Negatively charged LUV compartments layering on the inner surface of a positively charged GUV. Subcompartments have a 1% Rh-PE fluorescently labeled membrane. (e) Positively charged LUV compartments layered on negative LUV compartment layers on the outer surface of a positively charged GUV. Negative and positive subcompartments have a 1% Cy5-PE or a 1% Rh-PE fluorescently labeled membrane, respectively. (f) Negatively charged LUV compartments layering on the inner and outer leaflets of a positively charged GUV. Inner and outer subcompartment layers have a 1% Rh-PE or a 1% Cy5-PE fluorescently labeled membrane, respectively. All experiments performed with 0.5 M sucrose (as the internal) and 0.5 M glucose (as the external) solutions. Green channel: calcein. Red channel: Rh-PE. Blue channel: Cy5-PE.

We use variations in salt concentration in the artificial cell’s environment as a trigger for assembling and disassembling compartments outside and inside an artificial cell, this way controlling their spatial organization. We have chosen 5–50 μm positively charged giant unilamellar vesicles (GUVs) as the artificial cell chassis and organelle-like negatively charged 100 nm large unilamellar vesicles (LUVs) as their subcompartments. Unless otherwise stated, the positive GUVs were composed of 10 mol % 1,2-dioleoyl-3-trimethylammonium-propane (DOTAP) and the negative LUVs were composed of 5 mol % 1,2-dioleoyl-*sn*-glycero-3-phospho-(1-rac-glycerol) (DOPG) (Figure 1a). Apart from charged lipids, our vesicles contained neutral bilayer forming lipids 90 mol % 1-palmitoyl-2-oleoyl-*sn*-glycero-3-phosphocholine (POPC) and 95 mol % 1,2-dioleoyl-*sn*-glycero-3-phosphocholine (DOPC) for GUVs and LUVs, respectively. Where fluorescently labeled lipids were used (Rh-PE or Cy5-PE), they were present at 1 mol %. We use these phospholipid formulations to produce nested vesicle structures where negatively charged compartments are attracted to positively charged artificial cell membranes (Figure 1b). At 5% DOTAP GUVs were not sufficiently charged for the compartment layers to form (Figure S1) and at 10% DOPG for LUVs there is a possibility of rare fusion events (vesicles become more fusogenic as the charged phospholipid concentration is increased). The compositions used are such that the vesicles have a low, nonfusogenic charged lipid concentration and their interactions should mainly lead to association, not fusion.^33,34^

We show that negatively charged compartments can coat the outer and inner surface of the positively charged membrane of the artificial cell in layers. Using this principle, we were able to engineer compartmentalized vesicle-based artificial cell chassis with several different architectures. By adding labeled LUVs to the external solution, we were able to generate artificial cells with vesicle compartments assembled on the external face of the membrane (Figure 1c). The high fluorescence intensity of encapsulated calcein indicates that the subcompartments are condensed and stable as there is no observed content leakage. By loading the GUVs with LUVs inside, we generated artificial cells with subcompartments layered on the internal face of the membrane (Figure 1d). We were able to manufacture artificial cells possessing two oppositely charged vesicle layers on the outward facing part of the artificial cell membrane by subjecting the artificial cell to two sequential layering events, first to deposit negatively charged compartments, followed by positively charged ones (Figure 1e). Finally, we produced artificial cells with vesicle compartments coating on both the internal and external faces of the membrane by having negatively charged LUVs both in the artificial cell interior and exterior (Figure 1f). The external compartment assemblies were created by mixing solutions of negative LUVs with solutions of positive GUVs followed by excess LUV removal via centrifugation. The inner compartment layers were created by encapsulating negative LUVs into GUVs using the phase transfer method.^35^ Details of methods used are described in the Experimental and Supporting Information sections. The negatively charged subcompartments formed layers in a uniform manner. When layering a positively charged LUV layer on negative LUV layers, it was noticed that the 10% DOTAP compartment layers distributed in a patchy manner due to vesicle interaction with negatively charged BSA coating the imaging chambers (Figure 1e).^36,37^

Fluorescently labeled subcompartments became associated with the artificial cell surface through electrostatic interactions and ∼99% (*n* > 100 GUVs observed) of the artificial cells were coated with oppositely charged compartment layers. Next, we compare the fluorescence intensities of the GUV membrane to the fluorescence intensity of associated LUV layers (Figure S2). We show that the fluorescence intensity of the subcompartment layer is ∼4 times higher than the fluorescence intensity of the uncoated GUV membrane. In both cases 1 mol % Rh-PE was used to label the vesicles. This suggests that the subcompartments form a shell composed of interlinked LUV building blocks around the GUV.

**Figure 2:**
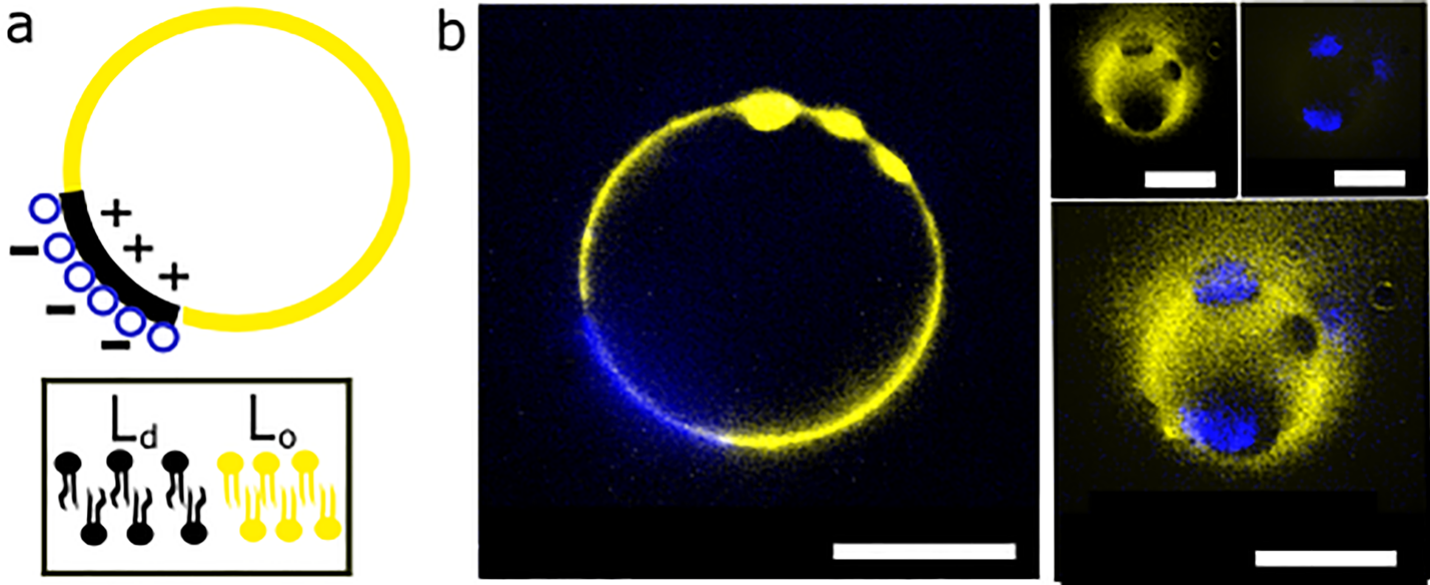
Charged subcompartments associate with phase separated artificial cells in patterns. (a) Schematic of oppositely charged vesicle association with charged membrane domains. (b) Confocal microscopy images of phase separated artificial cells with subcompartments associated with phase separated domains. Different focal planes of the artificial cell membrane are shown: equatorial focus (left) and polar focus (right). Subcompartments have a 1% Cy5-PE fluorescently labeled membrane. Artificial cells have a 2% NBD-PE fluorescently labeled membrane. Yellow channel: NBD-PE. Blue channel: Cy5-PE. Scale bar is 10 μm.

To add further complexity to our structures, we have also incorporated this architectural motif into charged phase separated GUVs composed of a fluorescently labeled quaternary mixtures of 33 mol % DPPC (1,2-dipalmitoyl-*sn*-glycero-3-phosphocholine), 38 mol % DOPC, 10 mol % DOTAP, 16.7 mol % cholesterol, and 2% NBD-PE. Partitioning of fluorescent lipids in different domains allows us to visualise them.^38^ This way, we assemble charged giant vesicles with coexisting liquid ordered and liquid disordered domains, reminiscent of the lipid rafts that are theorized to exist in biological cell membranes.^39^ In our case the membrane domains form before any interactions with charged vesicles and the liquid disordered domains appear as dark spots on the GUV surface (Figure S3). When negatively charged subcompartments are added to these phase separated GUVs, they associate with the domains, thus creating a patterned subcompartment layering architecture (Figure 2). On the basis of the positioning of associated oppositely charged subcompartments clusters, we can infer that the charged phospholipids in these giant vesicles membranes pool into the phase seperated domains (Figure 2b). When we focus in and out of the artificial cell focal plane, we observe that the subcompartments are colocalized on the dark nonlabeled artificial cell membrane domains (Figure 2b). The imperfect subcompartment and domain localization during imaging is due to fast domain diffusion across the membrane.

### Salt-Mediated Reversible Outer Subcompartment Layer Disassembly

**Figure 3:**
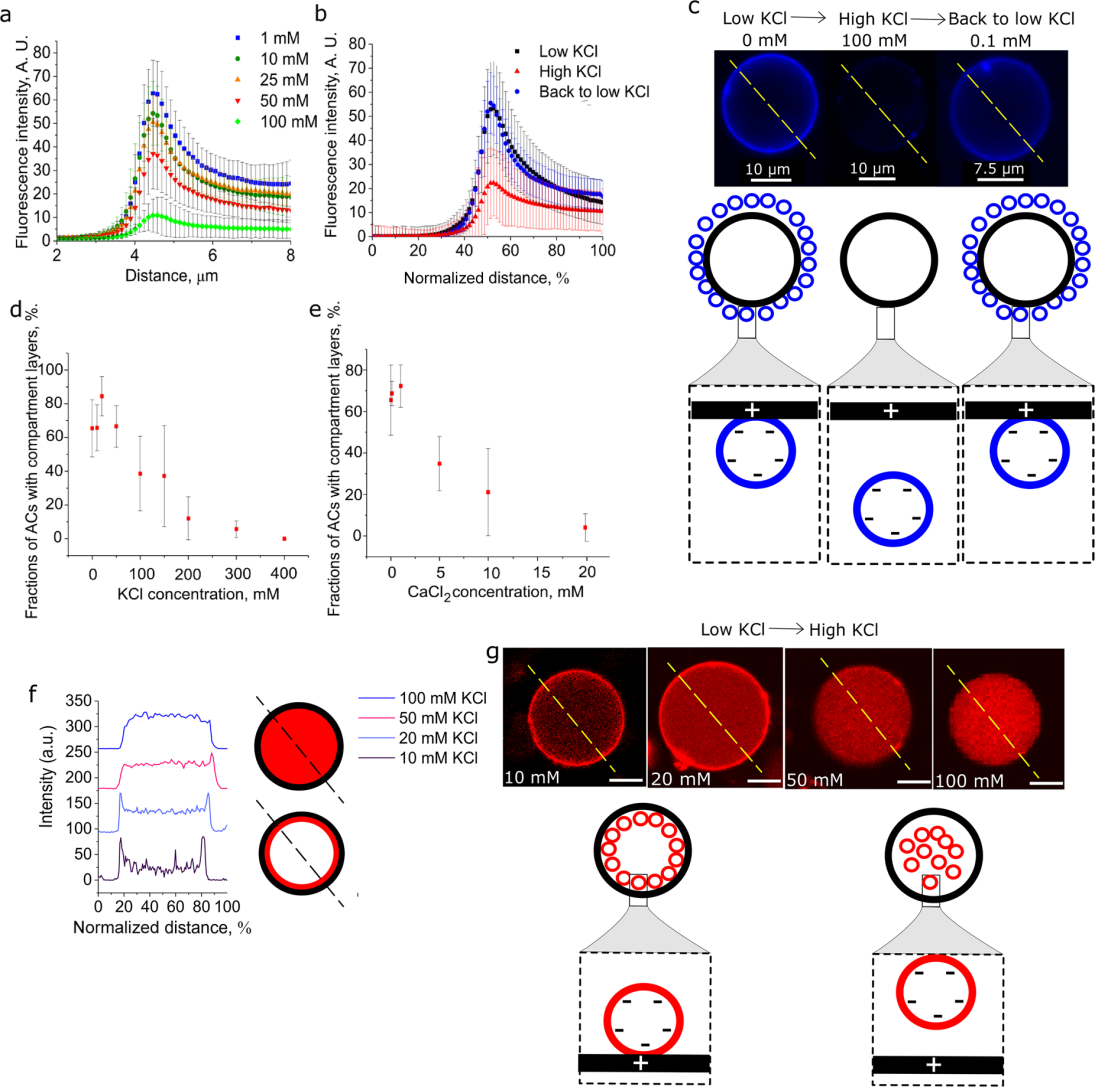
Salt-induced subcompartment release from artificial cell (AC) membranes. Fluorescence intensity profiles of the AC membrane edge of: (a) Negatively charged compartment layers on positive artificial cell surface in presence in varied amounts of KCl concentration (*N* > 17 for all these experiments); subcompartments are loaded with calcein dye. (b) Negatively charged compartment layers on the artificial cell in the presence of low (0 mM), high (100 mM), and back to low (0.1 mM) KCl concentrations. Subcompartments have a 1% Cy5-PE fluorescently labeled membrane. (c) Confocal microscopy images of negative compartment layers on positive artificial cell surface when the structures are transferred from low, to high, and back to low salt concentration conditions. Subcompartments have a 1% Cy5-PE fluorescently labeled membrane. This results in layer release and recoating with compartment layers (GUVs measured *N* > 33 for all conditions, experiment repeated *N* = 3). (d, e) Fractions of artificial cells that have compartment layers on their inner membrane in the presence of various concentrations of KCl or CaCl_2_, *N* = 3; (f) Fluorescence intensity profiles of negative inner compartment layers when artificial cell internal and external solutions contain various KCl concentrations. (g) Confocal microscopy images of negative compartment layers on positive artificial cells when the inner and outer artificial cell solution contains various KCl concentrations (yellow dotted lines show the area used for compartment layer fluorescence intensity profile measurements in panel f). Subcompartments have a 1% Rh-PE fluorescently labeled membrane. All experiments performed with 0.5 M sucrose (as the internal) and 0.5 M glucose (as the external) solutions. Error bars are standard deviations, scale bar for g is 10 μm. Red channel: Rh-PE. Blue channel: Cy5-PE.

The electrostatic nature of the membrane/membrane interactions enabled us to controllably disperse the externally layered structures by increasing the surrounding salt concentration. When we increase the outer KCl concentration from 1 to 100 mM, the compartment layers disassociate from the outer artificial cell membrane due to electrostatic screening of the attractive forces (Figure 3a and Figure S4). In order to assess if the drastic change in fluorescence localization was due to interaction of the dye with salt, we evaluated calcein fluorescence intensity for the salt conditions we used (Figure S5a). We found that when KCl concentration is above 100 mM, the fluorescence intensity only decreased by 20%. We also found that fluorescence intensity of the lipid probes remain stable when in the presence of 0–400 mM KCl and 0–20 mM CaCl_2_ (Figure S5). We thus conclude that these fluorescent dyes are suitable for our experiments. This dispersal process is reversible: multicompartment layers can be built up again by lowering the outer salt concentration back to ∼0.1 mM (Figure 3b,c). This way, the coated artificial cell is restored and can be used for repeated salt-triggered outer compartment layer dispersal and reassembly

### Dependence of Inner Subcompartment Layer Assembly on Salt

Next, we demonstrate that *inner* compartment layer assembly was influenced by the salt concentration in the GUV lumen (Figure 3d–g, Figures S6 and S7). We generated positively charged GUVs with negatively charged LUVs encapsulated inside, with a sweep of different salt concentrations coencapsulated in the GUV. We tested the effect of KCl (10–400 mM KCl; Figure 3d and Figure S6) and 0.1–20 mM CaCl_2_ (Figure 3e and Figure S7). We found that as the concentration of salt increased, the proportion of GUVs with assembled LUVs layers decreased (Figure 3f). Selected examples of this gradual disassembly are shown in Figure 3g. Both K^+^ and Ca^2+^ influence the inner layer formation by screening the electrostatic attractive forces. However, as Ca^2+^ ions are divalent, the salt concentration threshold at which layering was not observed (20 mM) was lower than for monovalent K^+^ ions (200 mM). At these concentrations, the majority of the artificial cells had compartments homogeneously distributed in their interiors (96 ± 7.1% for 20 mM Ca^2+^; 87 ± 12.7% for 200 mM K^+^). Potentially, these differences could also be attributed to the ability of Ca^2+^ to strongly interact with the slightly negative POPC phospholipids comprising the artificial cell membrane (90%) shielding the inner artificial cell membrane from negatively charged compartments.^40^ It is important to note that calcium ions have been associated with vesicle fusion in PC/PS (phosphatidylserine) vesicles and not PG/PC vesicles, therefore we rule out the possibility of calcium-induced vesicle fusion in our system.^40−46^

### Salt-Mediated Inner Subcompartment Layer Disassembly

**Figure 4:**
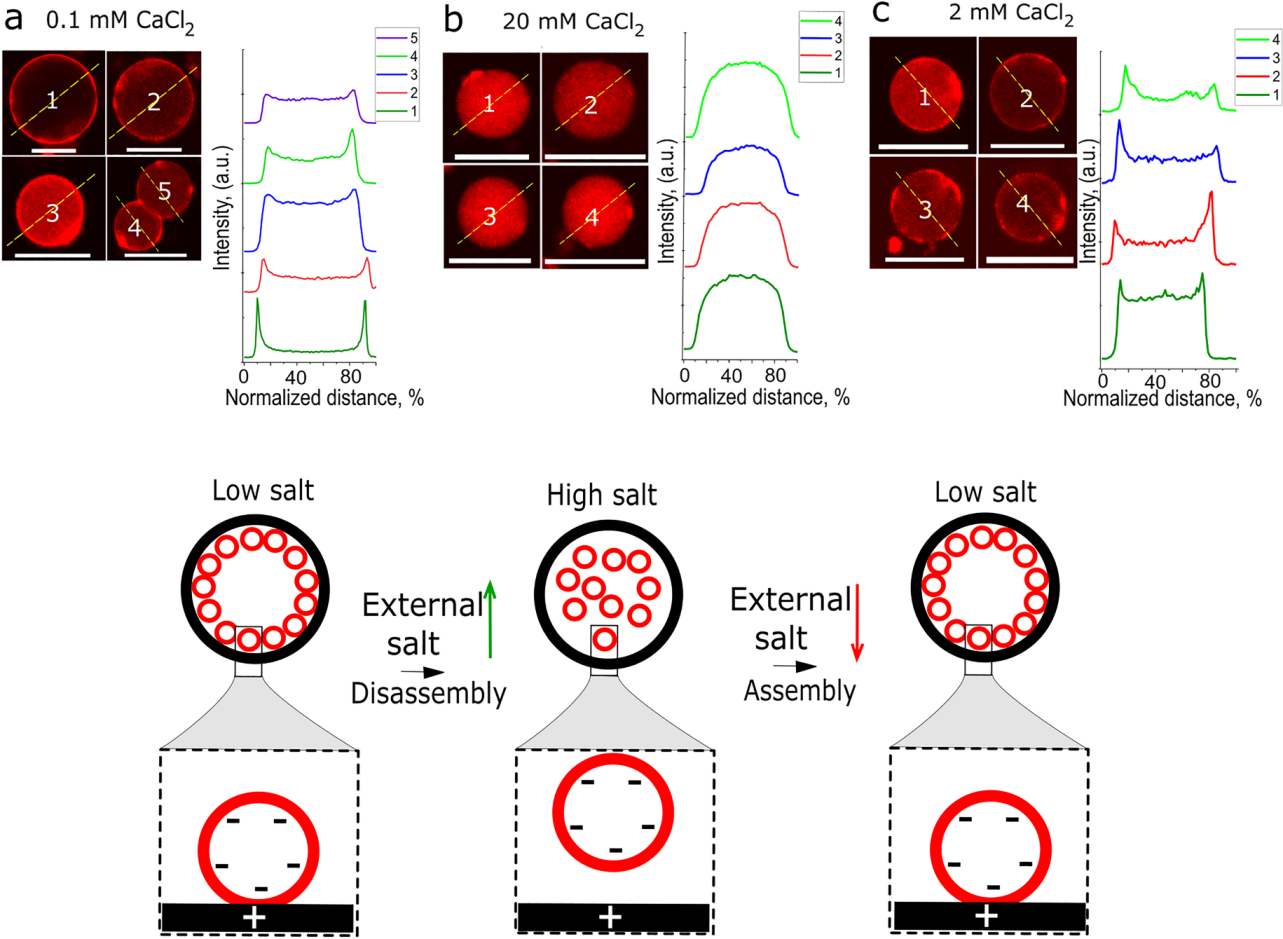
Subcompartment layer assembly and disassembly by changing the artificial cell external salt concentrations. Confocal fluorescence images and their representative fluorescence intensity profiles of positively charged artificial cells with negative subcompartment layers when artificial cells are placed in different CaCl_2_ solutions: (a) in low CaCl_2_ concentration, where the compartments form layers on the inner artificial cell membrane; (b) after transfer from low to high CaCl_2_ concentration, where the layers are disassembled; (c) after transfer from high back to low CaCl_2_ concentration, where the compartment layers are restored. Subcompartments have a 1% Rh-PE fluorescently labeled membrane. The measured distance is normalized for different AC diameters. All artificial cell transfers to various salt concentrations were conducted in glucose solutions. Red channel: Rh-PE. Scale bars are 10 μm.

We next demonstrated that by changing the artificial cell outer salt concentration we can trigger the release of their inner compartment layers (Figure 4, Figure S8). This showed that changes in the external environment led to a reconfiguration of the spatial arrangements of internal subcompartments. When artificial cells with inner compartment layers were transferred from low to high KCl or CaCl_2_ concentration solutions, the inner compartment layers disassembled and dispersed in the artificial cell lumen (Figure 4a,b and Figure S8). The subcompartment layers (external and internal) disassemble within a minute after the artificial cells are transferred into a higher salt concentration solution. Next, we tested if subcompartments could switch back to their original spatial organization once the external solution salt concentration was back to low salt conditions. We found that with KCl, reorganization was not reversible: once the layers were disassembled through the addition of external salt, they could not be reassembled when the concentration of external salt was reduced. When CaCl_2_ was used to modulate the subcompartment spatial organization, we achieved improved results compared to KCl: a higher fraction of artificial cells reversed to an inner compartment condensed phase, although, the statistical difference between high salt and low salt was not significant (Figure 4b,c and Figures S8 and S9).

We investigated if ion exchange during artificial cell transfer from low to high salt concentration solutions was responsible for inner LUV layer disassembly. We used a membrane-impermeable Fluo-3 pentapotassium salt and MQAE (*N*-(ethoxycarbonylmethyl)-6-methoxyquinolinium bromide) ion indicators for the detection of Ca^2+^ ions and Cl^–^, respectively^47,48^ (Figure S10). We show that when GUVs are transferred from low to high CaCl_2_ concentration solutions, the fluo-3 fluorescence does not increase, meaning that there was not a detectible influx of Ca^2+^ into the GUV. Moreover, MQAE fluorescence intensity did not decrease significantly when GUVs were transferred from low to high KCl concentration solutions, showing there was no detectable Cl^–^ influx that would quench the fluorescent probe significantly. These results show that the influx of CaCl_2_ or KCl into the GUV did not initiate the inner LUV layer disassembly. A calcein leakage assay was conducted where calcein-loaded GUVs were transferred from low to high salt concentration solutions (Figure S10). The GUV inner calcein fluorescence intensity did not change significantly during this experiment. For high salt concentration solutions, we used the maximum KCl concentration of 200 mM at which the GUVs are stable after transfer, demonstrating that GUVs were not leaky during our experiments. For CaCl_2_ we used 20 mM, as at this concentration, the GUV-LUV layers disassemble completely. We thus conclude that the inner salt concentration does not change during GUV transfer from low to high salt concentration solutions, suggesting that there is a different mechanism at play here. We speculate that this could be related to the GUV membrane capacitance: a change in the electrostatic charge characteristics of the outer leaflet upon addition of salt could potentially lead to altered vesicle electrostatic interactions with the inner leaflet.^49^

We observed that LUV fluorescence intensity decreased with decreasing LUV concentration meaning we could quantify the degree of compartment layering by comparing the fluorescence of LUVs in layers to the LUVs in the lumen (Figure S9a). The higher the salt concentration, the closer the fluorescence intensity mean ratio values are to 1, meaning that LUVs are being dispersed into the artificial cell lumen (Figure S9b,c). We use this membrane/lumen fluorescence ratio to evaluate how successfully the artificial cell changed states between assembled and disassembled compartments.

### Comparing KCl and CaCl_2_ Influence on Inner Artificial Cell Subcompartment Layering

Artificial cells with inner compartment layers are more sensitive to environmental changes of CaCl_2_ concentration than KCl which we attribute to the increased valency of Ca^2+^ compared to K^+^. If we compare LUV fluorescence intensity ratios to the fractions of artificial cells with layers at each described condition, we find the compartment layers reform more effectively in CaCl_2_ environments that in KCl (Figure S8), but the recoated compartment layers are not as uniform as before the salt concentrations changes (Figure 4a,c and Figure S9d,e). The difference on how both KCl and CaCl_2_ can modulate the layering of negatively charged compartments can be analyzed by comparing the change in membrane/lumen (M/L) fluorescence ratio with increasing KCl/CaCl_2_ concentration (Figure S11). We observe a plateauing of M/L ratio toward 1 at significantly decreased CaCl_2_ concentrations compared to KCl, in a similar manner to results shown in Figure 3d,e and Figure S8, indicating increased sensitivity of artificial cell compartment organization to the presence of external Ca^2+^ versus K^+^, respectively. By applying four-parameter logistic fits (commonly used to model the response of a biological system to a drug^50^) to both data sets, a dose–response relationship can be established between external salt concentration and M/L ratio (compartment layering) that estimates the onset salt concentration for disassembly. This allows us to quantify the difference in artificial cell layering response to different salts, using the salt concentration that produces a 50% reduction in M/L ratio (EC50) as a comparative measure. We have found that the system shifts from LUV layer assembly to disassembly at 59.46 ± 9.49 mM and 6.24 ± 7.1 mM for KCl and CaCl_2_, respectively. Here, the salt acts as a repressor (for compartment layer formation) and comparing EC50K^+^ with EC50Ca^2+^ indicates that the multicompartment artificial cells designed here are 10 times more sensitive to changes of environmental CaCl_2_ concentrations than KCl. These findings could relate to the reported ability of Ca^2+^ ions to restrict lipid mobility and become integral parts of negatively charged membranes composed of PS or PG phospholipids as these ions have nonspecific binding sites with charged phospholipid moieties (phosphate group in particular).^40,51^ It is reported that monovalent ions such as Na^+^ do not have a tendency to insert themselves into the membrane.^51^ This could explain why negatively charged compartment membranes are more sensitive to Ca^2+^ local concentrations than K^+^ or Na^+^. Our findings support using CaCl_2_ to rapidly switch between compartment layer assembly/disassembly and indicate that similar artificial cells could respond even more sensitively to higher valency metal salts. As artificial cell response here is determined by electrostatic interactions between the GUV and LUV membranes, varying the composition of GUV and LUVs and using different ions (e.g., Fe^3+^, Mn^5+^) should enable the generation of artificial cells with distinct onset conditions, facilitating simple methods to control different artificial cell populations present in the same environment with a single external ionic trigger.^52^

### Mechanical Switch for Inner Subcompartment Layer Disassembly

**Figure 5:**
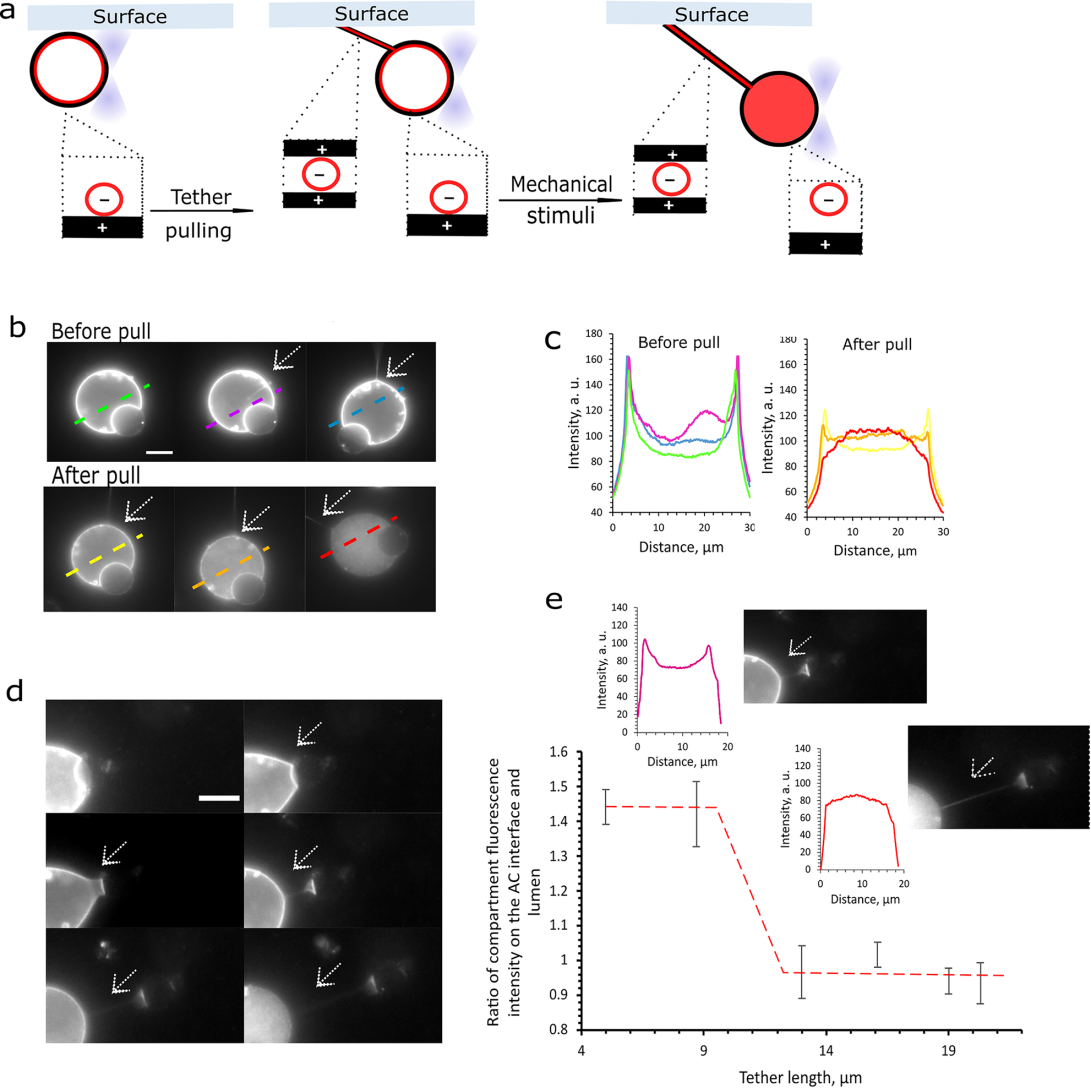
Charged artificial cell membrane responds to membrane mechano-stimuli by releasing their inner subcompartment layers. (a) Pulling an artificial cell membrane tether connected to the imaging well surface (or to a another GUV). Subcompartments appear to colocalize on the inner artificial cell membrane and within the tether. Tether pulling induces artificial cell inner subcompartment layer disassembly; (b) Fluorescence microscopy images of tether formation and pulling using optical traps; (c) Fluorescence intensity profiles of subcompartment layers disassembling upon tether pulling in panel b; (d) Fluorescence microscopy images of an artificial cell segment being pulled, and a membrane tether forming connecting the two structures. With increasing tether length, the inner subcompartment layers disassemble; (e) Fluorescence intensity profiles of subcompartment layers, showing disassembly during tether pulling and the dependence of subcompartment layering and tether length. The layer disassembly is expressed in the fluorescence intensity ratios of compartments colocalized on the membrane and in the lumen (M/L). With increasing membrane tether length, the subcompartments transition from a condensed to a dispersed state. Subcompartments have a 1% Rh-PE fluorescently labeled membrane. The red dashed line is included to guide the eye. Outer solution of 0.1 M KCl and 0.4 M glucose used in all these experiments. Scale bars are 10 μm.

Next, we will consider using mechanical forces to gain control over artificial cell compartmentalization. We show that mechanical stimuli can be used to switch the artificial cell compartmentalization from a layered state to a dispersed state (Figure 5). Mechanical changes across the artificial cell membrane are induced by pulling a membrane tether (Figure 5a) using optical tweezers.^53^ Tethers are formed by pulling the vesicles away from a surface to which they adhere (Figure 5a,b). Interestingly, the fluorescently labeled subcompartments colocalize not only on the inner artificial cell surface but also within the membrane tether (Figure 5b). Further pulling of the tether results in the disassembly of inner artificial cell subcompartment layers. In Figure 5b,c fluorescently labeled subcompartment distribution within the cell during tether pulling is shown in selected fluorescence microscopy images. After tether pulling, the sharp fluorescence peaks representing the layers on the artificial cell inner surface decrease while the subcompartment fluorescence intensity in the artificial cell lumen grows, indicating subcompartment disassociation (Figure 5c).

To better evaluate how tether length influences subcompartment dissociation, we have tested another way to form membrane tethers. Because of electrostatic interactions, GUVs that have been brought together using optical tweezers can adhere to one another and form a double bilayer at their contact site, also known as a VIM (vesicle interface membrane).^54^ When the vesicles are being pulled away from one another a membrane tether between them forms.^54^ In our case, we used optical tweezers to detach a small part of the GUV, thus creating a membrane tether in between these two structures (Figure 5a,d). In both tether formation scenarios, the formed membrane tether is composed purely of the lipid bilayers of the artificial cell (Figure 5a). We evaluated how increasing the membrane tether length affected the subcompartment inner distribution, expressed by their fluorescence M/L ratio as described previously. When the membrane tether length is increased from 5 to 13 μm the artificial cell compartmentalization state is switched from layered to disperse subcompartments (Figure 5e). This process was irreversible and pulling the tether further did not impact artificial cell stability. This switchable feature creates an additional level of control over subcompartment spatial organization. It has been previously reported that tether immobilization on the GUV surface alone can cause rearrangements of its membrane domains suggesting that tether pulling could have an impact on GUV membrane phospholipid arrangements.^55^ With that in mind, we speculate that during tether pulling charged phospholipids might segregate to the tether away from the artificial cell membrane. This process could occur with the help of tether bound charged subcompartments strongly interacting with the cell membrane lipids. As the tether is pulled, the subcompartments sequester the charged membrane lipids and as a result lowering their concentration across the artificial cell membrane. Charge depletion across the inner artificial cell membrane in turn would cause the gradual disassembly of artificial cell subcompartments layers. An alternative hypothesis is that assembly/disassembly is dictated by changes in membrane tension as the membrane is being pulled.^56,57^

### Further Characterization of Artificial Cells with Compartment Layers

We have investigated how negatively charged compartment layers influence the artificial cell membrane fluidity. For this purpose, we have measured the generalized polarization (GP) of Laurdan (6-dodecanoyl-2-dimethylaminonaphthalene)^58^ incorporated into the artificial cell membrane when the artificial cell has associated compartments on its outer membrane. This probe changes its fluorescence emission wavelengths in response to changes in phospholipid packing (435 nm for ordered packing in a gel state and 500 nm for unordered packing in a fluid state) and this is used for Laurdan GP value calculations.^58^ We have shown that the Laurdan GP value for artificial cell membranes does significantly (*p* < 0.05) decrease once in the presence of negative vesicle layers, suggesting that the artificial cell membrane becomes more fluid (Figure S12). This suggests that the compartment layers interact with the membrane. These results are in accordance with previous findings where LUVs with a higher mol % of charged lipids led to a strong LUV association with the GUV outer membrane that promoted GUV bursting.^34^ In our system, we use a charged lipid fraction that enables electrostatic interactions that are strong enough for association but too weak to disrupt the integrity of the artificial cell chassis. This is critical to using dynamic compartment condensation in future applications: disruption of subcompartment membranes would lead to uncontrolled content leakage or destruction of the spatial organization of the artificial cell.

## Conclusion

We describe an artificial cell system that allows the spatial organization of artificial cell subcompartments to be modulated in response to chemical and mechanical triggers. Depending on the setup, subcompartments can switch between condensed layers on the membrane surface, to being dispersed in the cell interior or the surrounding environment. Our system relies on electrostatic interactions between membranes, which means that it can be modulated using salts. We show that switching from a condensed to a dispersed state can be modulated by using both KCl or CaCl_2_, with a concentration dependent relationship. The greater amount of salt present, the more the screening, and the more the dispersed state is favored. We also find that divalent cations are more effective at shielding the charge and are therefore better modulators of this phenomenon. With CaCl_2_, the assembly and disassembly has potential to be reversible, with the cells able to switch between the two states. KCl was found to be sufficient for reversible outer layer release from the artificial cell surface leading to compartments released to the environment. Tether pulling using optical tweezers- can act as a mechanical stimulus and trigger artifical cell inner subcompartment layer disassembly on a single-cell level. This is likely due to a charged lipid depletion occurring during membrane tether pulling which results in reduced attractive forces favoring subcompartment association. These findings show that we can control artificial cell dynamic compartmentalization using chemical and mechanical stimuli on a population and single-cell levels.

Our work describes ways to achieve closer mimicking of dynamic compartmentalization events in biological cells (e.g., modulation of organelle contact sites with other organelles, proteins or substrate pools, and membraneless organelle formation in response to cellular stress). This design concept can also be used as a signal transduction module in artificial cell engineering, for example to regulate the internal biochemistry in the cell in response to environmental cues (e.g., if a change in the level of compartmentalization can be coupled to change in function or behavior). Moreover, layered structures we generate can be exploited as a platform for cell–cell communication (e.g., through the release of exosome-like structures from the cell surface), and for localized release of therapeutic cargo in delivery applications.

Control over compartment spatial organization could enable researchers to create artificial cells with compartments arranged in the most optimal way for desired product production, for example by pooling the compartments with related functions together, this way lowering the distance for bioactive molecule transport from one compartment to another. An analogy can be drawn with phase separated structures (both in membranes^59^ and in the cytosol^60,61^) that have biological importance and have been exploited to give artificial cells extended functionality.^60,62,63^ Importantly, our system does not rely on protein machinery. Instead, it relies on biomembrane design principles for dynamic remodelling of compartment organization, which is further evidence that membrane biophysical principles can be a useful tool in the arsenal for the construction of functional synthetic cell systems.

## Experimental Section

### Materials

All lipids were purchased from Avanti Polar Lipids (Alabaster, AL) as powders and used without further purification. Lipids used include DOTAP (catalog number 890890), DOPG (catalog number 840475), DOPC (catalog number 850375), POPC (catalog number 850457), DPPC (catalog number 850355), cholesterol (catalog number 700100), 18:1 Rh-PE (catalog number 810150), 18:1 Cy5-PE (catalog number 810335), and 16:0 NBD-PE (catalog number 810144). Lipid mixtures were prepared by codissolving the required molar ratios of lipids in chloroform. Fluorescence probes Fluo-3 (catalog number F3715) and MQAE (catalog number E3101) was purchased from Thermofisher scientific (Waltham, MA, USA). Also, 150 nm AuNPs (catalog number 746649), BSA (≥99% assay, catalog number A4161), calcein (catalog number C0875), Sephadex G-50 affinity column (catalog number G5080), and all buffer reagents were purchased from Sigma-Aldrich (Gillingham, UK).

### Generating Artificial Cells with Different Hierarchical Architectures

Lipid films for compartment (large unilamellar vesicle (LUV)) and artificial cell (giant unilamellar vesicle (GUV)) formation were prepared by dissolving DOTAP, POPC, DOPG, and DOPC in chloroform to a stock concentration of 25 mg/mL. For each desired phospholipid composition aliquots of these stocks were transferred into glass vials followed by evaporating the chloroform with a nitrogen stream and desiccation overnight. For LUV extrusion DOPG:DOPC mol % 5:95 lipid films were rehydrated with 0.5 M glucose (for outer layering) or 0.5 M sucrose (for inner layering) and different salt concentration solutions to a lipid concentration of 10 mg/mL. The solutions were gently vortexed for 2 min and extruded through a 100 nm membrane. In the case where calcein was encapsulated into LUVs, the solutions underwent 4 freeze–thaw cycles followed by extrusion. The unencapsulated calcein was then removed using a Sephadex G50 affinity column.

GUVs were prepared using the phase transfer method. Ten milligrams of DOTAP:POPC mol % 10:90 were sonicated in 1 mL of mineral oil for 1 h. When GUVs were used to compare their membrane fluorescence intensity to LUV layer fluorescence intensity, 1 mol % Rh-PE was added to the phospholipid film. In the case of phase separated GUVs 10 mg mol % 33:38:10:16.7:2 of DPPC, DOPC, DOTAP, cholesterol, and NBD-PE was used. In an Eppendorf tube 200 μL of a lipid in mineral oil solution was layered on top of 20 μL of 0.5 M sucrose or 4 mg/mL LUVs in 0.5 M sucrose if inner LUV layers are formed. The mixture was gently pipetted up and down up to 20 times. The created water-in-oil emulsion was layered on 300 μL of 0.5 M glucose. The sample was centrifuged for 30 min at 10,000 rfc. The formed GUVs formed a pellet on the bottom of the Eppendorf tube and the supernatant and mineral oil layer were removed. The GUV pellet was washed with 300 μL of 0.5 M glucose and centrifuged again for 10 min at 9,000 rcf speed. The supernatant was removed, and the washed GUVs were resuspended in 200–300 μL of a glucose solution.

For LUV layer formation on the GUV surface, 30 μL of negative LUVs (DOPG:DOPC:18:1 Liss Rhod PE mol % 5:94:1) was mixed with 150 μL of positive GUVs (DOTAP:POPC mol % 10:90) and incubated for 5 min (all in 0.5 M glucose). The excess LUVs were removed by pelleting the GUVs by centrifugation and removing the supernatant. The GUV pellet was then dissolved in 0.5 M glucose.

For the LUV layer comprising negative and positive LUVs formation on the GUV surface, 300 μL of 10% DOTAP GUVs was mixed with 60 μL of negative LUVs (DOPG:DOPC:18:1 Cy5-DOPE mol % 5:94:1) and incubated for 5 min followed by excess LUV removal by centrifugation. The GUVs with DOPG LUV layers were then mixed with 30 μL of positive LUVs (DOTAP:DOPC:18:1 Liss Rhod PE mol % 10:89:1) and incubated for 30 min. The excess LUVs were removed using centrifugation and the pelleted GUVs were resuspended in 0.5 M glucose. A summarized scheme of the procedures used is shown in Figure S13.

For LUV layer formation on both inner and outer GUV membranes, 4 mg/mL LUVs (DOPG:DOPC:18:1 Liss Rhod PE mol % 5:94:1) were encapsulated into GUVs using the phase transfer method. Here, 150 μL of the formed GUVs with inner negative LUV layers was mixed with 30 μL of negative LUVs (DOPG:DOPC:18:1 Cy5-DOPE mol % 5:94:1) and incubated for 5 min. The excess LUVs were removed using centrifugation.

The prepared samples with different artificial cell architectures were loaded into BSA-coated imaging chambers and visualized using a Leica TCS SP5 II inverted confocal microscope. Images were analyzed using ImageJ software.

### The Release and Relayering of Outer Artificial Cells Compartment Layers

For 18:1 Cy5-DOPE labeled or calcein loaded LUV disassociation from the GUV outer membrane, the GUV and LUV mixture was diluted up to 1.5 mL with 100 mM KCl 0.5 M glucose solution and centrifuged for 10 min at 6,000 rfc speed. After GUVs sediment to the bottom of the Eppendorf tube, the supernatant is removed, and the GUVs are dispersed into 100 mM KCl 0.5 M glucose solution to its original volume (180 μL). For LUVs to be relayered on the GUV surface, the same procedure is applied with 0.5 M glucose. To test if LUV layer release corresponds to increasing outer solution salt concentration, the described process was repeated using an outer solution KCl concentration range of 1–100 mM in 0.5 M glucose. The LUV layer fluorescence intensity was evaluated by confocal microscopy (Cy5 651/670 nm, calcein 495/515 nm). The summary of the procedures is depicted in Figure S14.

### The Release and Relayering of Inner Artificial Cells Compartment Layers

For 18:1 Liss Rhod PE labeled LUV disassociation from the GUV inner membrane, the GUVs with LUV layers were transferred from their primary low salt 0.5 M glucose solution (20 mM KCl or 0.1 mM CaCl_2_) to high salt glucose solutions (0.32 mM glucose and 200 mM KCl or 0.5 M glucose and 20 mM CaCl_2_). After we recorded the inner LUV layering changes, the GUVs were then transferred from high salt glucose solution back to a low salt 0.5 M glucose solution (20 mM KCl or 2 mM CaCl_2_). Artificial cells and compartment layers were visualized using confocal microscopy (556/580 nm) and the images were analyzed using ImageJ software.

### Artificial Cell Tether Pulling for Inner Subcompartment Release

The GUV tether experiments were conducted using an inverted Nikon fluorescence microscope. 150 nm AuNPs were added to the vesicles at a 1:9 ratio. Vesicle’s membrane tethers were formed using procedures described in Bolognesi et al.^54^ GUVs were moved by focusing a laser of 100 mW (at trap) or lower on their interface. Membrane tethers were formed with 0.1 M NaCl in the external glucose solution. The membrane tether length was measured by ImageJ.

### AC Compartment Fluorescence Intensity Profile Measurements

Using ImageJ software, fluorescence intensity profiles and means were measured. First, the image background fluorescence was subtracted, and then 8 μm length (image scale) and 20 arbitrary units’ thick lines were drawn on 5 different areas of the LUV fluorescent shell. Using these lines, the fluorescence intensity profiles were produced. ImageJ tools were also used to measure the fluorescence intensity means of LUV layer and LUVs in the lumen.

### Estimating the Partitioning of Subcompartments in the Artificial Cell

After confirming that LUV fluorescence (1% 18:1 Liss rhod PE) in the GUV lumen varied linearly with concentration in the ranges encapsulated (Figure S9a), we compared fluorescence values at the membrane (defined by a measurement range of *x* μm) to those in the center of the vesicle lumen for KCl and CaCl_2_ gradients (10–100 mM and 0.1–20 mM, respectively). The membrane/lumen ratio parameter is then defined as
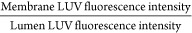
1where an M/L ratio >1 indicates localization of LUVs at the GUV membrane compared to bulk fluorescence in the GUV lumen, an M/L ratio ∼1 indicates equal fluorescence at the membrane and in the GUV lumen, while an M/L ratio <1 would indicate partitioning of the LUVs to the core of the GUV. In this study, we expect the M/L ratios to vary between >1 to ∼1 due to the electrostatic interaction between the GUV and LUV membranes employed.

### Ca^2+^ and Cl^–^ Influx/Outflux Evaluation with Fluoresceent Probes Fluo-3 and MQAE

These experiments were conducted using an inverted Nikon fluorescence microscope. GUVs were loaded with 4 mg/mL LUVs and 100 μM Fluo-3 or 10 mM MQAE for either Ca^2+^ or Cl^–^ detection. A control of GUVs with 100 μM Fluo-3 and 20 mM CaCl_2_ was included. The Fluo-3, 4 mg/mL LUVs and 500 mM sucrose loaded GUVs were transferred from 500 mM glucose to 20 mM CaCl_2_ 500 mM glucose solutions and the fluorescence intensity of Fluo-3 (λ_ex_ = 506 nm, λ_em_ = 526 nm) inside GUVs was imaged before and after 1 h of incubation using a FITC filter. MQAE and 4 mg/mL LUVs and 500 mM sucrose loaded GUVs were transferred from a 500 mM glucose solution to a 200 mM KCl 300 mM glucose solution, and MQAE fluorescence intensity (λ_ex_ = 319 nm, λ_em_ = 462 nm) within GUVs was imaged using the DAPI filter. The vesicles were imaged inside a quartz imaging chamber, and a control of GUV loaded with MQAE and 200 mM KCl was added for comparison. The fluorescent ion probe fluorescence intensities were measures using ImageJ.

### Calcein-Leakage Assay in GUVs

These experiments were conducted using an inverted Nikon fluorescence microscope. GUVs were loaded with 4 mg/mL LUVs, 20 mM KCl, 500 mM sucrose, and 10 mM calcein. The GUVs were transferred from a 500 mM glucose solution to a 200 mM KCl in 300 mM glucose solution. Fluorescence images of calcein-loaded GUVs (λ_ex_ = 495 nm, λ_em_ = 515 nm) were taken (FITC filter), and the GUV inner calcein fluorescence intensity was measured using ImageJ.

### Subcompartment Influence to AC Fluidity Using Laurdan Reagent

For the Laurdan assay, previously published methods^49^ were used as references. Laurdan stock in dimethylsulfoxide was mixed with a 0.3 mM 10% DOTAP GUV solution in 0.5 M glucose to a final dye concentration of 0.45 μM. The mixture was incubated for 10 min, during which Laurdan incorporates into the GUV membrane with a molar ratio of 1:670 of Laurdan molecules to GUV phospholipids. Laurdan-labeled GUVs were mixed with 5% DOPG LUVs and incubated for 10 min. The fluorescence measurements were performed in a Cary Eclipse Fluorimeter (Agilent Technologies, USA). The Laurdan fluorescence intensities at 435 and 500 nm were measured by exciting the probe with a 350 nm wavelength. The generalized polarization (GP) of Laurdan in GUVs with and without outer LUV layers was calculated as follows
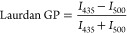
2where *I*_435_ is the Laurdan fluorescence intensity when the probe is in an ordered lipid packing environment in gel-phase and *I*_500_ is the Laurdan fluorescence intensity in an unordered lipid packing in fluid-phase. An increase of the Laurdan GP value corresponds to a shift toward the physical properties of a gel-like membrane, while a decrease represents a shift toward properties of a fluid-like membrane.

## Supporting Information Available

Additional confocal microscopy images of LUV coated GUVs, phase separated GUVs; analysis on how different salts affect the LUV coating on the inner/outer GUV; results on the LUV influence on GUV membrane fluidity; schemes depicting experimental methods (PDF)
Sub-compartment disassociation initiated by pulling a tether from the artificial cell surface (AVI)

## Supplementary Material

nn2c02195_si_001.pdf

nn2c02195_si_002.avi
