# Supplementary material for: Dynamic Reconfiguration of Subcompartment Architectures in Artificial Cells
Source: ACS Nano. 2022 Jun 13;16(6):9389–400. doi: 10.1021/acsnano.2c02195 (PMC9245354; doi:10.1021/acsnano.2c02195)
Supplement: Supplementary file 1 — nn2c02195_si_001.pdf [file nn2c02195_si_001.pdf]

Supporting Information

Dynamic Reconfiguration of Subcompartment Architectures in Artificial Cells

Greta Zubaite [1,2,3], James W. Hindley [1,2,3], Oscar Ces [1,2,3] and Yuval Elani* [2,4]

[1] Department of Chemistry, Molecular Sciences Research Hub, Imperial College London, London W12 0BZ, UK

[2] fabriCELL, Molecular Sciences Research Hub, Imperial College London, London W12 0BZ, UK

[3] Institute of Chemical Biology, Molecular Sciences Research Hub, Imperial College London, London W12 0BZ, UK

[4] Department of Chemical Engineering, Exhibition Road, Imperial College London, SW7 2AZ, UK

* y.elani@imperial.ac.uk

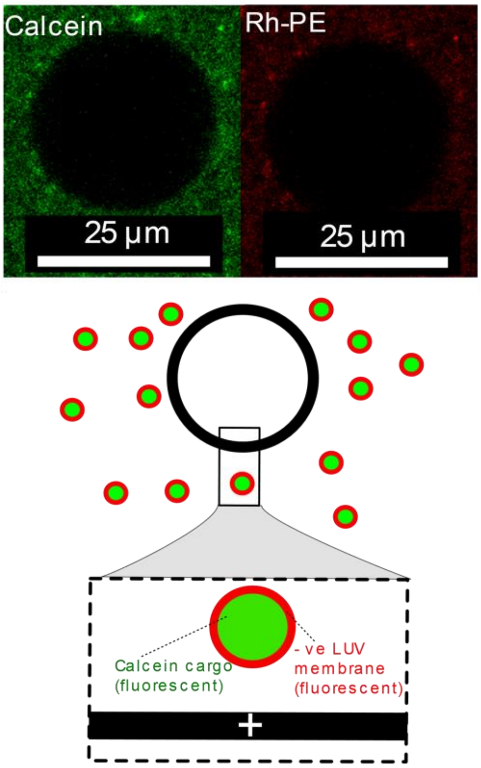

Fig. S1. 5% DOPG sub-compartment layers do not form on artificial cell outer comprised of 5% DOTAP: confocal microscopy images of LUVs loaded with calcein and labelled with 1% Rh-PE.

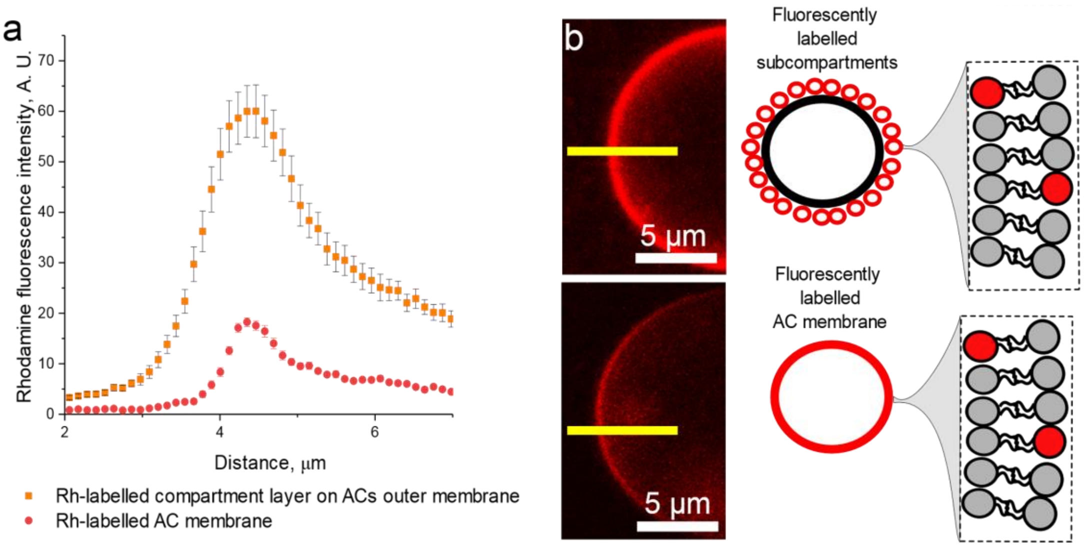

Fig. S2. Fluorescence intensity profile comparison (a) of 1% Rh-PE labelled negatively charged sub-compartment (5% DOPG LUVs) layering on positive artificial cell membranes (10% DOTAP GUVs) and 1% Rh-PE labelled GUV membrane. (b) Corresponding confocal microscopy images and schematics to graph (a). The error bars are standard errors. For both sets each GUV (N = 13) was measured at 5 different areas.

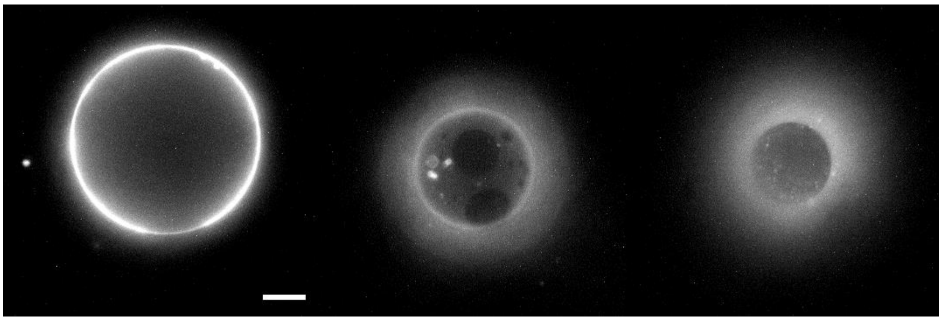

Fig. S3. Fluorescence images of positively charged phase separated GUVs. GUV membrane domains were imaged on different focal planes in order to detect phase separated domains. GUV membrane was labelled with Rh-PE lipids. Scalebar is 10 μm.

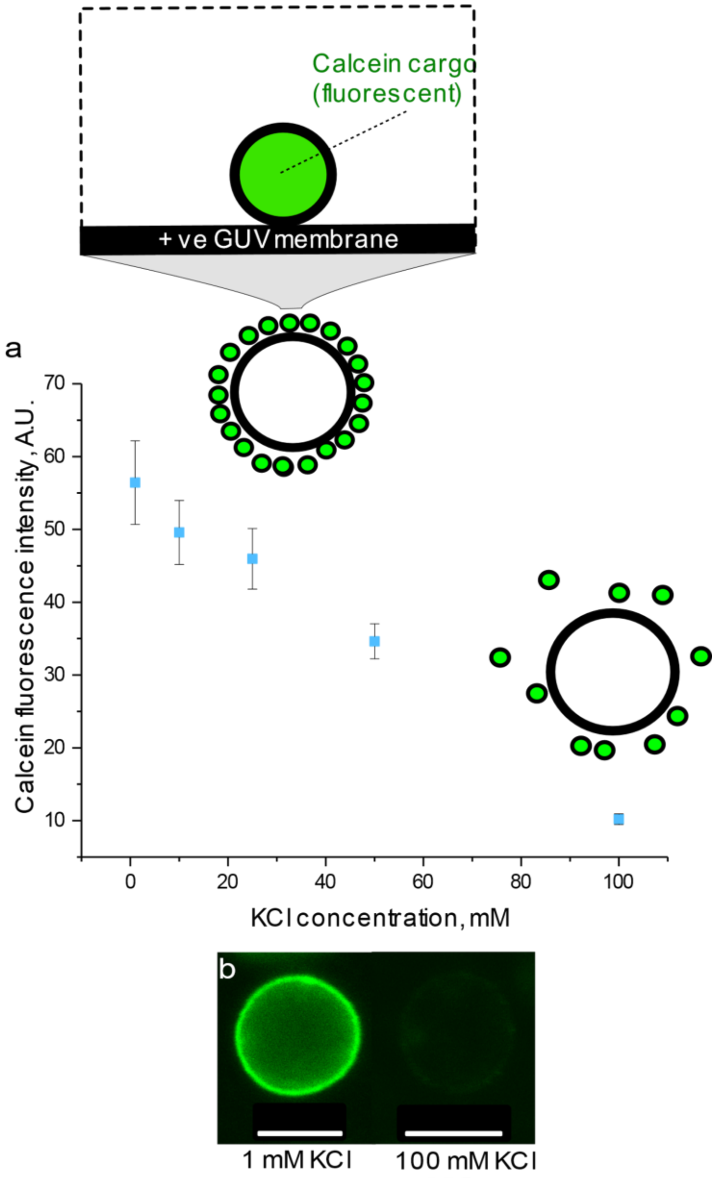

Fig. S4. Artificial cell outer compartment layers disassemble with increasing external salt concentration. (a) The fluorescence intensity values of sub-compartment layers across the artificial cell decrease linearly as calcein-loaded sub-compartments are released from the outer artificial cell membrane when the layers are exposed to 1 mM – 100 mM KCl. (b) Confocal microscopy images of sub-compartment layers in presence of low and high KCl concentrations. Error bars are standard errors, scale bars are 10 µm.

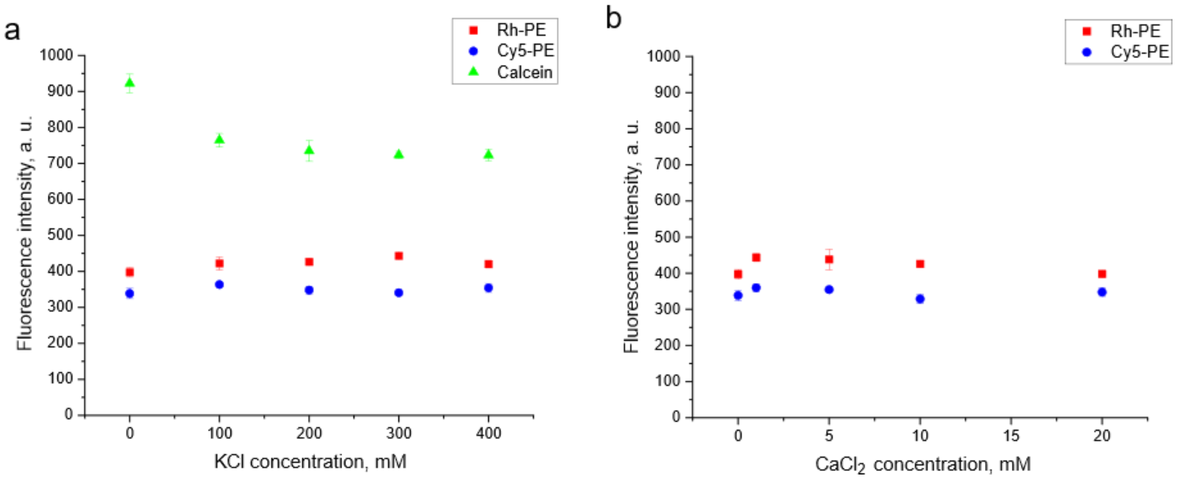

Fig. S5. Rhodamine-PE, Cy5-PE and calcein fluorescence intensity dependence on salt concentration. a) With increasing KCl concentration the fluorescence intensity of Rhodamine-PE and Cy5-PE remains stable, while calcein decreases by ~20% when the KCl concentration increases above 100 mM. b) with increasing $CaCl_2$ concentration the fluorescence intensity of Rhodamine-PE and Cy5-Pe does remains stable. Error bars are standard deviations.

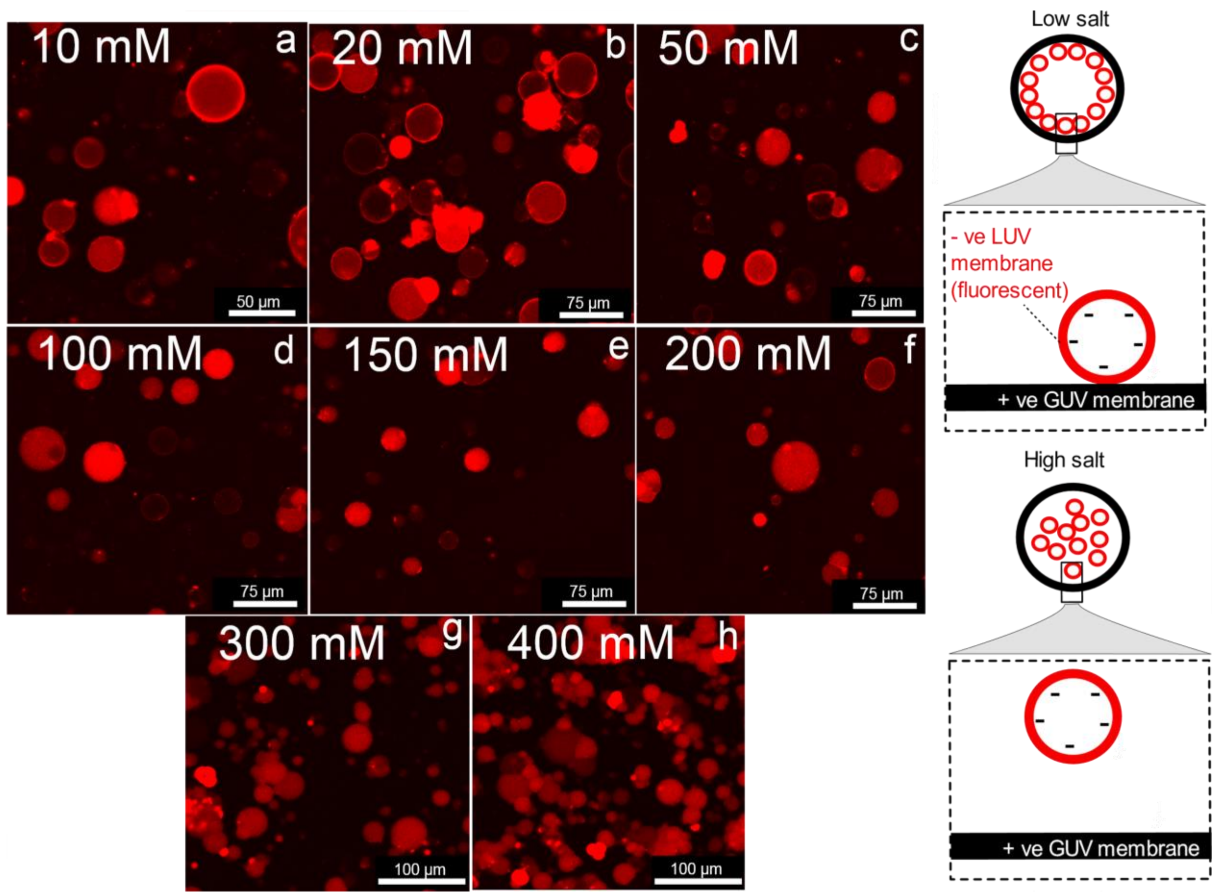

Fig. S6. Confocal fluorescence images of how various KCl concentrations affect the inner artificial cell compartment layering. This shows that increasing the inner and outer salt concentration leads to inner sub-compartment layer disassembly. Sub-compartment membranes were labelled with Rh-PE lipids.

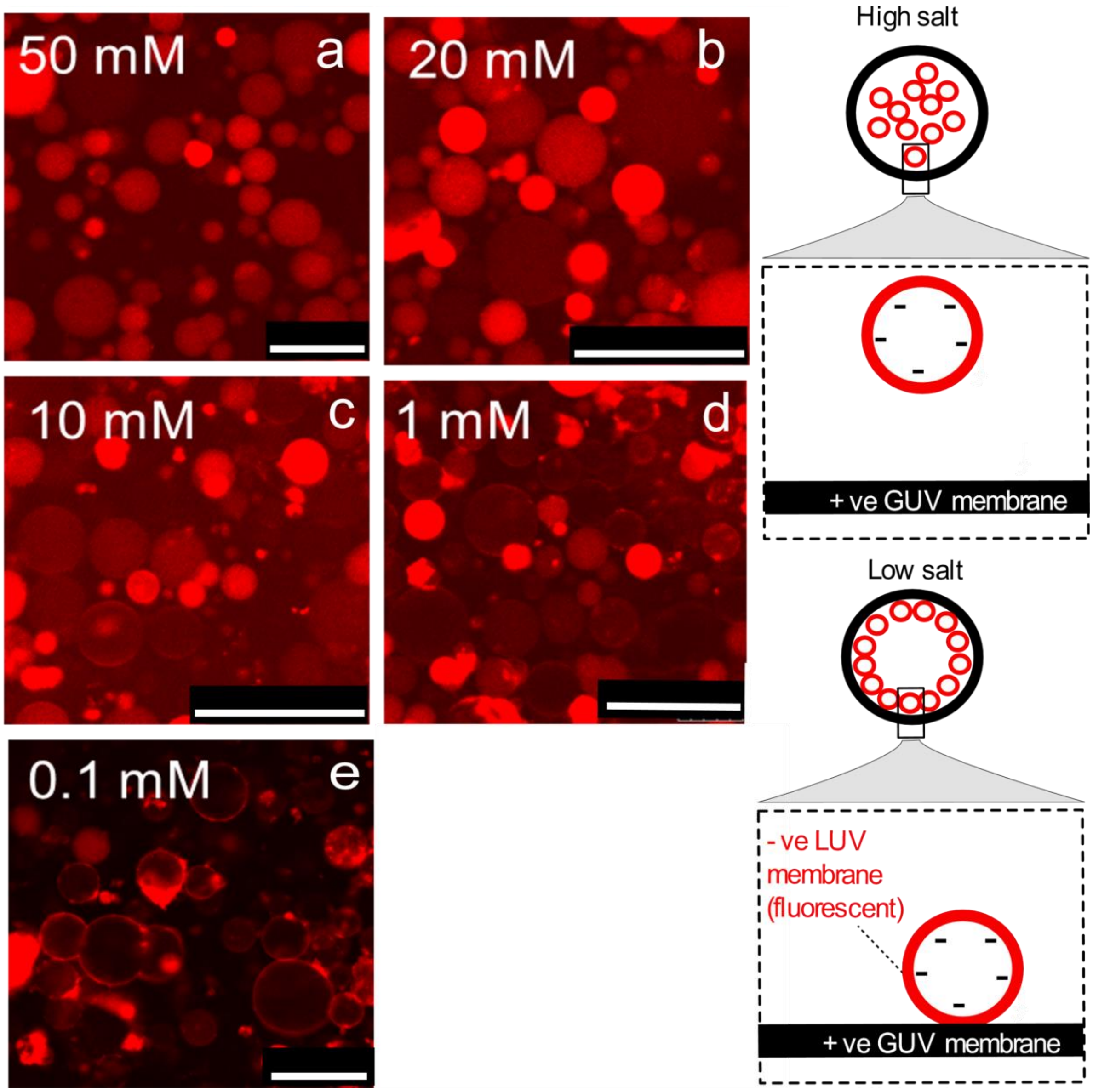

Fig. S7. Confocal fluorescence images of how various $CaCl_2$ concentrations affect the inner artificial cell compartment layering. This shows that increasing the inner and outer salt concentration leads to inner sub-compartment layer disassembly. Sub-compartment membranes were labelled with Rh-PE lipids. Scale bar 50 μm.

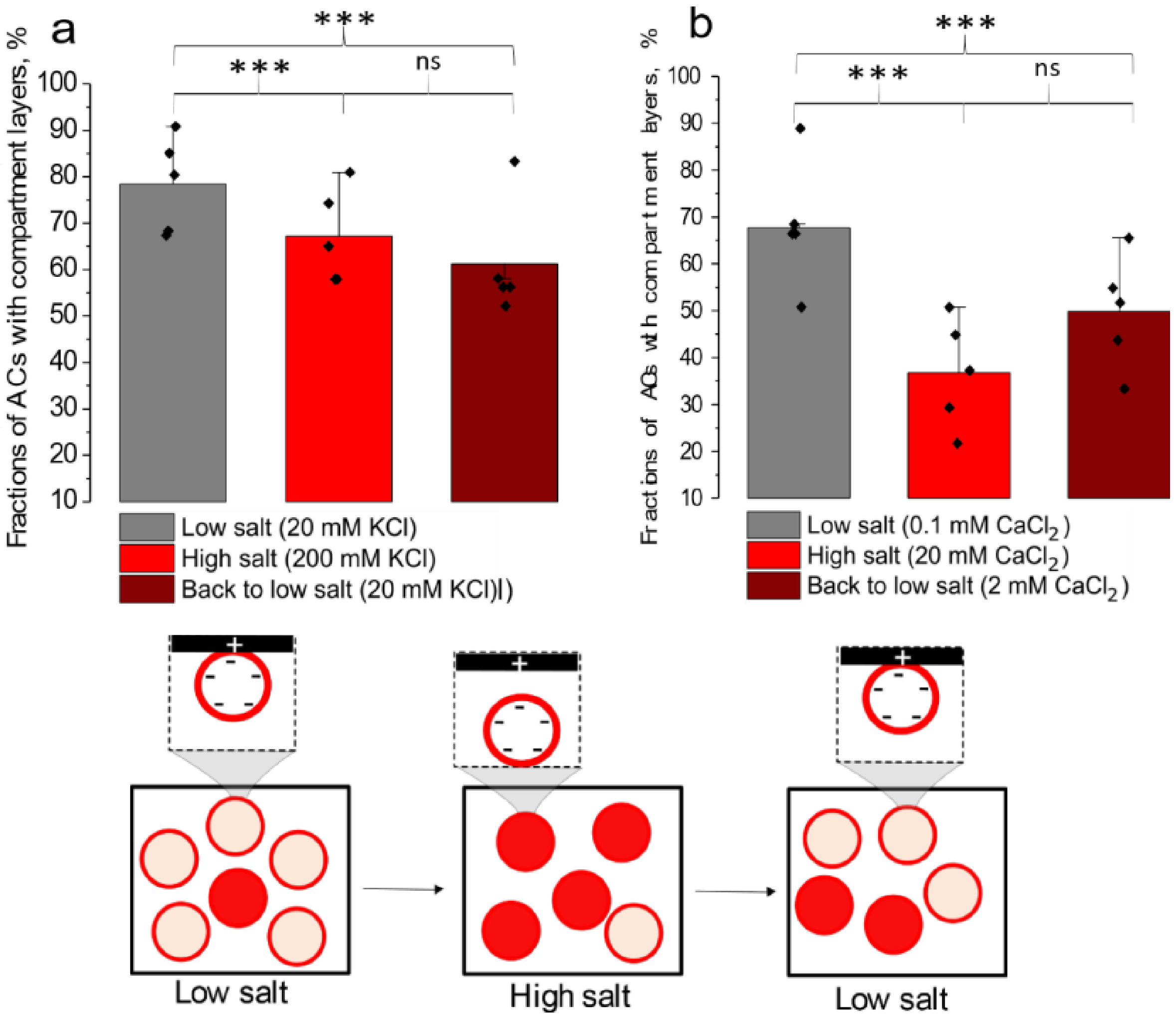

Fig. S8. Artificial cell inner compartment layer disassembly in response to the external KCl and CaCl2 concentration changes. Fractions when (a) the artificial cells are dispersed in low salt solution (proportion of GUVs with compartment layers 78.4 ± 10.3%), transferred to high salt solution (proportion of GUVs with compartment layers 67.2 ± 10.2%) and then transferred back to a low salt solution (proportion of GUVs with compartment layers 61.2 ± 12.5%); (b) of artificial cells with compartment layers when the artificial cells are dispersed in low salt solution (proportion of GUVs with compartment layers 67.7 ± 12.6), transferred to high salt solution (proportion of GUVs with compartment layers 36.8 ± 11.7%) and then transferred back to a low salt solution (proportion of GUVs with compartment layers 49.8 ± 12.1%). For (a,b) errors bars are standard deviations, N=5, for, N>90 GUVs were counted for these experiments.

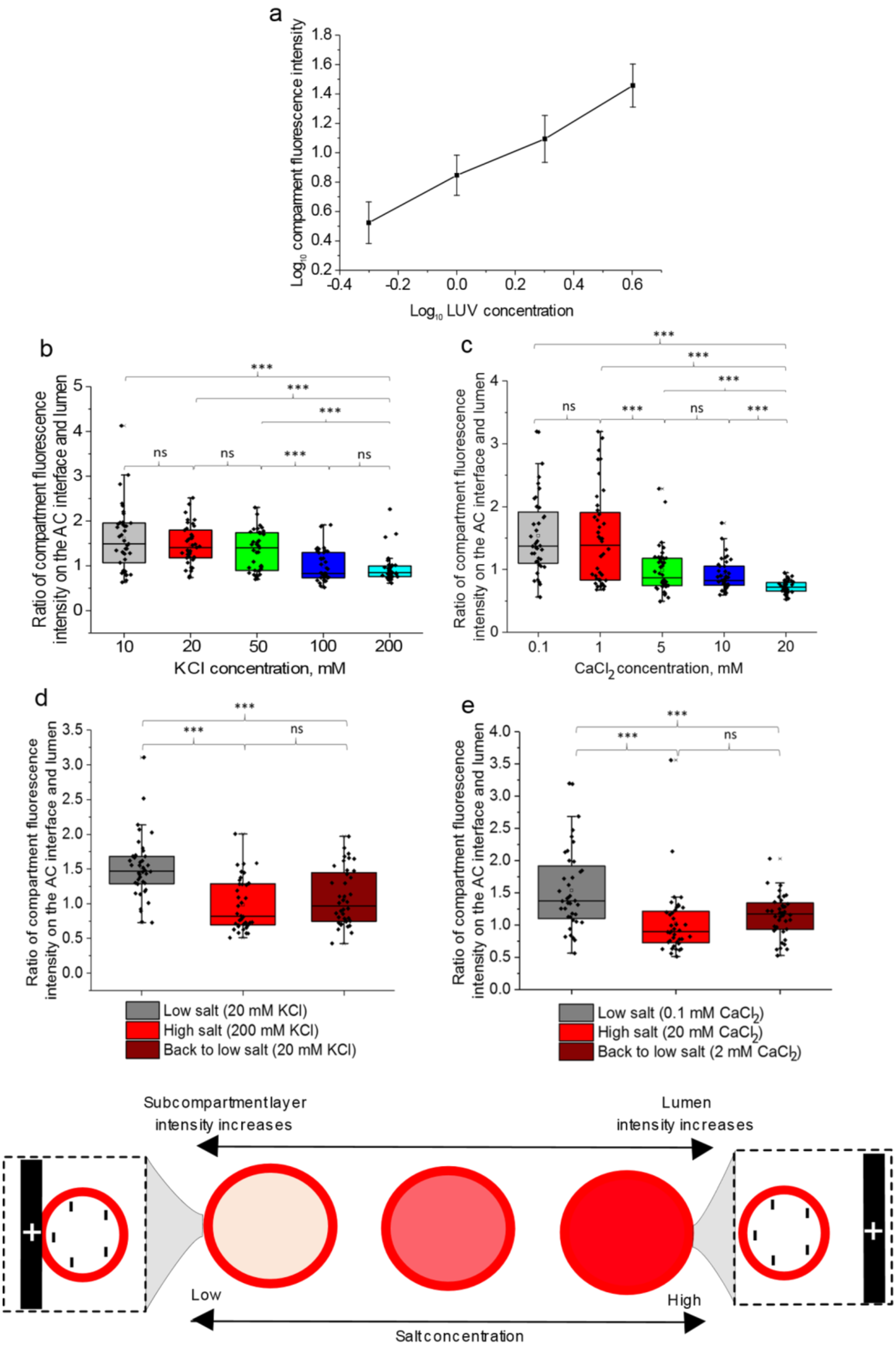

Fig. S9. Sub-compartment spatial localization inside the artificial cell described in the fluorescence intensity ratios of compartments localized in layers and in the lumen when in presence of different salt concentration solutions: (a) linear dependency of 1% Rh- PE labelled DOPC LUV fluorescence intensity on LUV concentration; (b, c) LUV layer and lumen fluorescence intensity ratios in presence of 10 – 200 mM KCl and 0.1 – 20 mM $CaCl_2$; (d, e) LUV layer and lumen fluorescence intensity ratios when artificial cells are in low salt conditions, then transferred into high salt conditions; then

followed by transfer back to low salt conditions. Box plot with quartiles, median, mean, N=39/40 for (b – e).

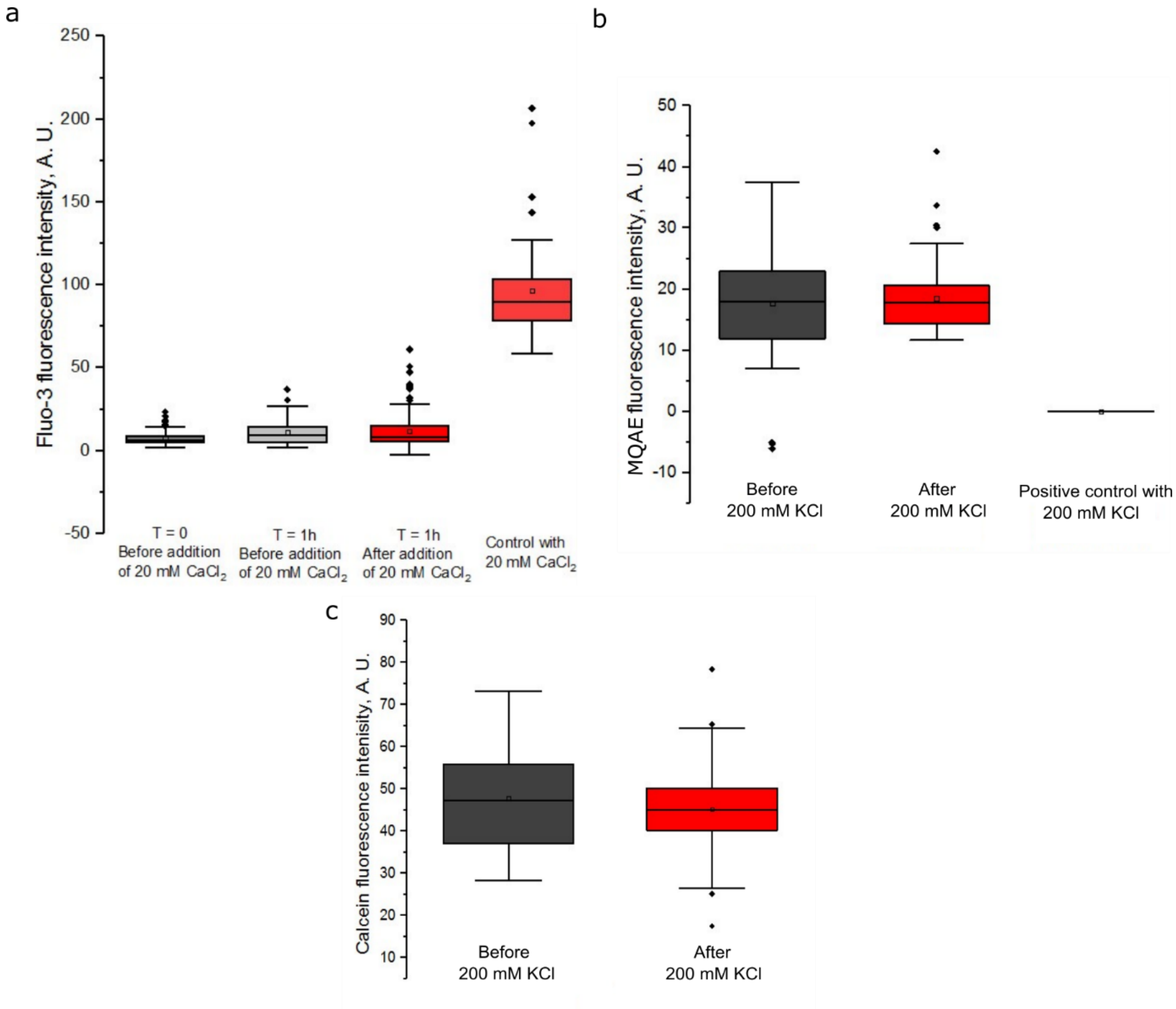

Fig. S10. The inner GUV Ca^{2+} and Cl^{-} concentration does not change and the GUV membrane is not leaky to water when GUVs are transferred to higher salt concentration solutions. In each GUV sample, except for controls, the inner salt concentration is 0 mM or 20 mM for $CaCl_2$ and 0 mM for KCl respectively. a) 100 µM of Fluo-3 fluorescent probe was used to evaluate that the inner GUV Ca^{2+} ion concentration did not changed during GUV transfer from 0 mM $CaCl_2$ to 20 mM $CaCl_2$. Fluo-3 fluorescence intensity increases when in presence of Ca^{2+} ions. The results were compared to a control of GUVs loaded with 20 mM $CaCl_2$ and 100 µM Fluo-3. b) 10 mM of MQAE fluorescent probe was used to evaluate that the inner GUV Cl^{-} ion concentration did not changed during GUV transfer from 0 mM KCl to 200 mM KCl. MQAE fluorescence intensity decreases when in presence of Cl^{-} ions. The results were compared to a control of GUVs loaded with 200 mM KCl and 10 mM MQAE probe. c) GUV stability evaluation using 10mM calcein leakage assay; inner GUV calcein fluorescence intensity does not change significantly when transferred from 0 mM KCl into 200 mM KCl concentration solution. We used 200 mM concentration of KCl at which the salt gradient does not effect the stability of the GUV. We used the maximum $CaCl_2$ concentration at which the highest fraction of GUV-LUV layers disassemble.The GUV osmotic pressure was balanced with glucose solution during each transfer to different salt concentrations. For (a-c) errors bars are standard deviations, N=3, for, N>50 GUVs were counted for these experiments.

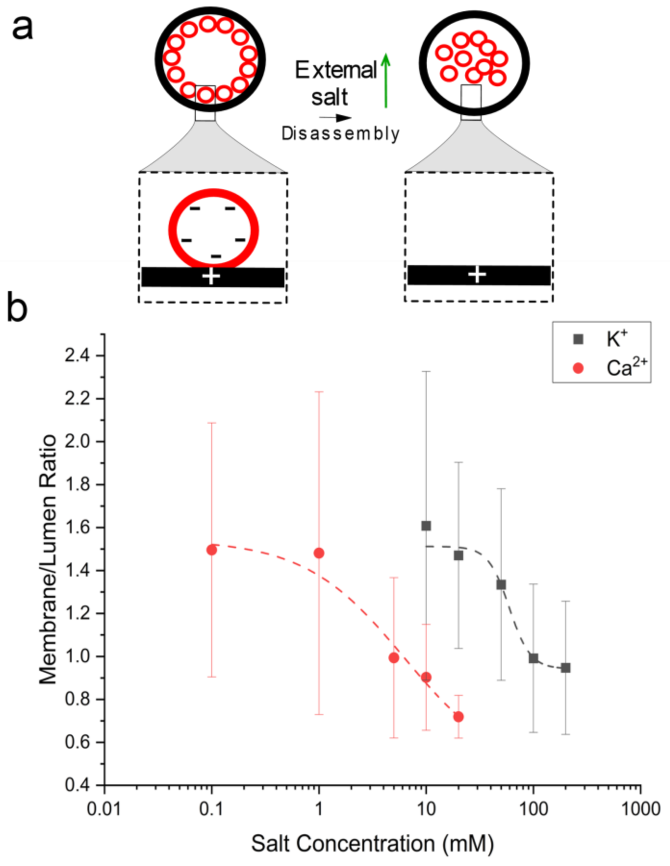

Fig. S11. Sub-compartment dissociation from the inner AC membrane with increasing salt concentration. Half maxima at which the system switches from layered assembly to layer disassembly are 59.46 ± 9.49 mM and 6.24 ± 7.10 mM for KCl and $CaCl_2$ respectively, as determined by logistic fits using OriginPro 2020 to both data sets (adj. r_2 = 0.942 / 0.929 for KCl and $CaCl_2$ respectively). Error bars = one standard deviation, N=39/40 for KCl and $CaCl_2$ respectively.

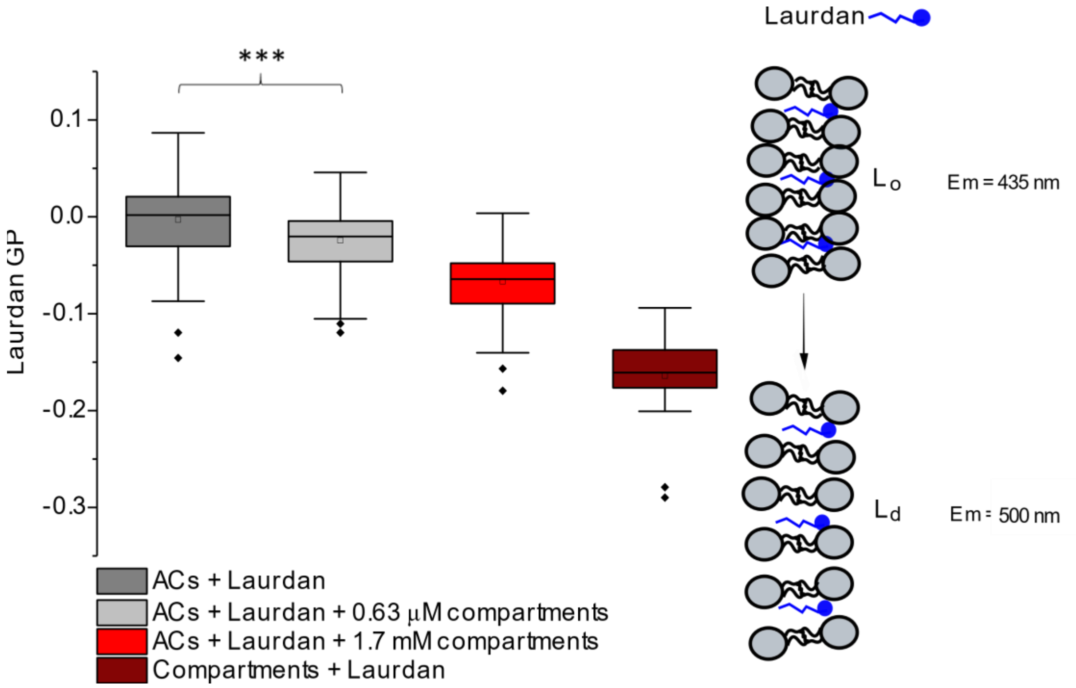

Fig. S12. Negatively charged sub-compartments increase the positively charged artificial cell membrane fluidity. Artificial cell outer membrane phospholipids packing characterized by Laurdan generalized polarization values for 10% DOTAP GUVs with 2.25 µM incorporated Laurdan in presence of 0.63 µM, 1.7 mM of 5% DOPG LUVs and for 5% DOPG LUVs labelled with Laurdan). The GP values for ACs + Laurdan with and without 0.63 µM LUVs are significantly different ($p = 0.0004$). Excited at 350 nm. Box plot with quartiles, median, mean and outliers, N=3.

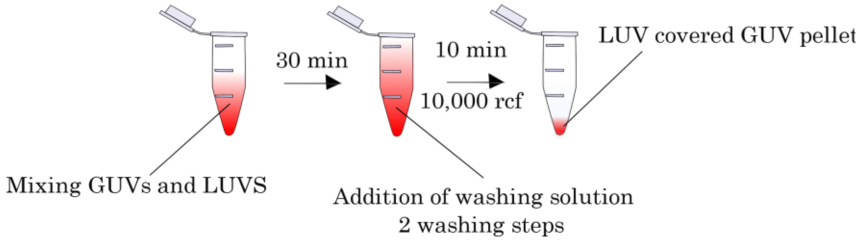

Fig. S13. Scheme depicting the preparation of artificial cells coated with outer compartment layers.

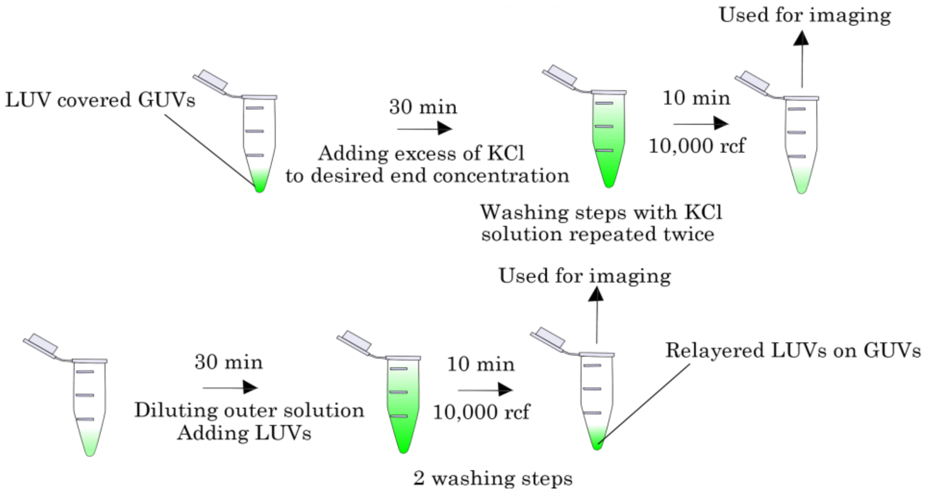

Fig. S14. Scheme depicting the release and re-layering of sub-compartments on the outer artificial cell membrane.

SI Video – Video showing us pulling a tether from an artificial cell with associated vesicle sub-compartments on the inner leaflet. As the tether is pulled, the inner vesicles transitions from a condensed state on the artificial cell surface, to a dispersed state in the cell lumen.
